# The job stress and subjective well-being among Chinese primary and secondary school teachers: the role of marital quality and social support

**DOI:** 10.3389/fpsyg.2025.1625960

**Published:** 2026-01-21

**Authors:** Xiaolong Wang, Zhenzhen Zhang, Feng Xu, Ranran Wang, Hongliu Ouyang

**Affiliations:** 1 College of Education Science, Kashgar University, Kashgar, China; 2 Kashi Municipal Bureau of Education, Kashgar, China; 3 Faculty of Education, East China Normal University, Shanghai, China; 4 College of Transportation, Kashgar University, Kashgar, China; 5 Faculty of Early Childhood Education, Sichuan Preschool Educators College, Mianyang, China; 6 Faculty of Human Development, Sultan Idris Education University, Tanjong Malim, Malaysia

**Keywords:** teacher job stress, subjective well-being, social support, marital quality, mediation

## Abstract

**Introduction:**

Although the relationships among job stress, subjective well-being, social support, and marital quality have been examined across various cultural contexts, limited research has addressed these mechanisms within the Chinese cultural setting. This study aimed to explore how social support and marital quality interact to explain the association between teachers’ job stress and subjective well-being.

**Methods:**

A total of 189 married primary and secondary school teachers from China participated in this study. Data were collected through validated self-report questionnaires measuring job stress, marital quality, social support, and subjective well-being. Structural equation modeling was used to test the hypothesized mediation model.

**Results:**

The findings indicated that marital quality served as a significant mediator in the relationship between job stress and subjective well-being. Furthermore, marital quality and social support jointly exerted a partial sequential mediating effect, suggesting that stress originating in the school context can cascade into family and social domains.

**Discussion:**

These results highlight the interdependent nature of teachers’ work and family systems and extend the ecological systems theory by demonstrating how interactions across school, family, and social networks contribute to teachers’ well-being. The findings underscore the importance of supportive marital and social environments in mitigating the negative impact of job stress.

## Introduction

Subjective well-being (SWB) has long been an important topic in psychology and education. SWB is generally defined as an individual’s overall cognitive evaluation of life satisfaction, as well as the presence of positive and negative emotions in daily life and work (Diener, 1984). A large body of research has shown that SWB is closely related to physical and mental health, social adjustment, and overall quality of life (Livingston et al., 2022; Gautam et al., 2024). For teachers, SWB not only concerns their own health and career sustainability but also directly affects teaching quality and student development (Nguyen-Thi et al., 2024; Assali, 2025).

Among the various factors influencing teacher well-being, job stress has consistently been regarded as a significant risk factor (Toshniwal and Narendran, 2020; Singh et al., 2023; Black et al., 2019). Empirical studies have shown that high levels of job stress lower life and job satisfaction and significantly increase the risk of emotional exhaustion (Mérida-López et al., 2022; Woloshyn et al., 2023), indicating that stress can undermine teachers’ subjective well-being. Research further indicates that teachers in China report relatively low levels of well-being (Ao et al., 2023). The combined burden of heavy teaching and administrative tasks, performance evaluations under the exam-oriented education system, and growing expectations from parents and society creates a work environment that is both highly demanding and stressful (Zhu and Zhai, 2025; Tang, 2023). Such conditions may be an important reason for the low levels of SWB among Chinese teachers. In this context, the exam-driven education system and the strong social expectations placed on teachers may further magnify the negative effects of job stress on their well-being.

Although previous studies have confirmed the adverse impact of job stress on teacher well-being, the mechanisms behind this relationship remain underexplored. Much of the existing literature has focused on individual psychological resources, such as self-efficacy (Hosseini et al., 2017), affective rumination (Sharma and Tiwari, 2025), and work engagement (Shao et al., 2025). However, teacher well-being is not only shaped by individual traits but also by social relational resources (Casinillo et al., 2020; Samosir and Idayani, 2022). In this regard, marital quality and social support represent two critical dimensions of relational resources that may serve as key pathways in understanding the link between job stress and well-being. Marital quality reflects the stability and emotional support provided within the family (Fincham and Linfield, 1997), while social support refers to perceived assistance from colleagues, friends, and broader social networks (Cohen and Wills, 1985). These resources may offer emotional compensation and buffering effects when teachers face occupational stress. Nevertheless, empirical studies that examine the roles of marital quality and social support in the stress–well-being relationship remain very limited, particularly within the Chinese educational and cultural context.

Building on this gap, the present study aims to systematically examine the relationship between job stress and SWB among teachers in China, with a particular focus on the mediating roles of marital quality and social support. By incorporating both family- and society-based relational resources, this study provides a deeper understanding of the mechanisms that shape teacher well-being. It also provides an empirical foundation for developing interventions and policies that enhance teacher well-being at multiple levels.

## The direct relationship between job stress and subjective well-being

Existing research has consistently shown a significant negative association between job stress and subjective well-being. In the Chinese educational context, Liao et al. (2023) found that higher levels of occupational stress among primary and secondary school teachers significantly reduced their life satisfaction and emotional well-being. Similarly, Zhu and Chang (2025) reported that university teachers experienced comparable outcomes, while Deng (2022) demonstrated that preschool teachers under long-term high pressure also exhibited substantially lower levels of subjective well-being. These findings collectively reveal that teachers across different educational stages are vulnerable to the erosive effects of job stress on well-being.

Beyond the education sector, similar patterns have been observed. Akgunduz et al. (2022) showed that job stress significantly reduced well-being among hotel employees in Turkey, and Lee and Park (2019) found that increased stress among Korean office workers was closely linked to declines in both well-being and overall quality of life. Together, these studies indicate that the adverse impact of job stress on well-being is a robust phenomenon across occupations and cultural contexts.

Despite this consistent evidence, the existing body of research remains limited in its sustained attention to the deeper processes and mechanisms underlying this relationship. In the unique educational and cultural context of China, teachers face long-term, high-intensity work demands and strong social expectations (Liao et al., 2023; Shao et al., 2025), which can further complicate how stress affects their well-being. Therefore, further investigation is necessary, particularly to explore the mechanisms through which job stress affects teachers’ subjective well-being.

## Marital quality as a mediator in the relationship between teachers’ job stress and subjective well-being

Marital quality generally refers to individuals’ overall evaluation of their marital relationship, encompassing satisfaction, functioning, and relational processes such as communication and conflict resolution (Fincham and Bradbury, 1987). Although direct evidence on the mediating role of marital quality in the relationship between teachers’ job stress and subjective well-being is scarce, prior research provides indirect support for this possibility. A large body of studies has consistently shown that individuals in satisfying marital relationships report higher happiness, fewer symptoms of anxiety or depression, and greater life satisfaction (Arya et al., 2024; Grover and Helliwell, 2019; Cheguvera and Dutt, 2021; Chełchowska, 2020). These findings highlight marital quality as a crucial relational factor closely tied to overall well-being. At the same time, research has documented links between occupational stress and marital functioning. Fallahchai et al. (2019) found that higher work stress was associated with lower marital quality, particularly among wives. Similarly, Soza-Herrera et al. (2022) reported a negative association between job stress and marital quality, whereas Commey-Mintah et al. (2023) observed a positive association in certain occupational contexts, suggesting that the relationship may be complex and context-dependent. Together, these studies indicate that job stress has the potential to influence marital quality, which in turn is closely related to well-being. This provides indirect evidence for considering marital quality as a potential mediator in the stress–well-being relationship.

From the ecological systems perspective (Bronfenbrenner, 1979, 2005), marital relationships are part of the microsystem and represent one of the most immediate environments shaping individual well-being. Stressors originating from the exosystem, such as heavy workload and accountability pressures, may spill over into family life, straining marital functioning and, in turn, undermining teachers’ SWB. Conversely, high marital quality can provide emotional security, interpersonal validation, and instrumental assistance that help teachers manage stress and sustain well-being. Despite these theoretical considerations, the mediating role of marital quality in the stress–well-being relationship has received limited empirical examination, particularly in non-Western educational contexts such as China.

## Social support as a mediator in the relationship between teachers’ job stress and subjective well-being

Social support is broadly defined as the perception that one is cared for, valued, and embedded in a supportive social network (Cobb, 1976). Although the mediating role of social support in the relationship between teachers’ job stress and subjective well-being remains underexplored, both theoretical considerations and empirical findings from related domains suggest its potential importance. Research in non-educational contexts has consistently demonstrated that social support functions as a mediator linking stressors to well-being outcomes. For instance, Yazdani et al. (2024) identified social support as a partial mediator between pandemic-related stress and life satisfaction, while Yıldırım and Green (2023) found that social support, alongside resilience, fully mediated the relationship between perceived stress and life satisfaction among university students. Similarly, Balcan (2023) showed that social support mediated the impact of operational stress on well-being in military personnel, underscoring its cross-contextual relevance as a protective mechanism.

Within the teaching profession, although direct tests of mediation are scarce, existing studies highlight that social support is strongly associated with teachers’ subjective well-being (Asfari et al., 2024; Fu et al., 2022; Mahasneh et al., 2024) and is also closely linked to their job stress (Maharani and Dinni, 2022; Tian and Isa, 2024). These findings suggest that teachers with higher social support experience greater well-being and lower stress, pointing to the possibility that social support may serve as a critical pathway through which job stress influences SWB.

From an ecological systems perspective (Bronfenbrenner, 1979, 2005), job stress originating from the exosystem may undermine teachers’ ability to perceive or mobilize support, while strong social support in the microsystem can help sustain emotional stability and buffer the adverse effects of stress. Thus, examining the mediating role of social support offers valuable insights into how relational contexts shape teachers’ well-being in high-pressure educational environments.

## The chain mediation of marital quality and social support

Beyond their independent roles, marital quality and social support may also function sequentially, reflecting mesosystem processes in which different microsystems interact. Prior research has shown that individuals in high-quality marital relationships are more likely to perceive and mobilize external support, as emotional intimacy and mutual trust within marriage facilitate broader interpersonal engagement (Mo et al., 2022; Xue and Jiang, 2023). In addition, supportive marital contexts can enhance individuals’ willingness to seek and utilize help from friends, colleagues, or community members, thereby expanding their social resource networks (Bhandare and Simon, 2024). These findings suggest that marital quality may facilitate the mobilization of social support, supporting the plausibility of a sequential pathway from marital quality to social support.

In line with the ecological systems framework, this perspective can be extended to the mesosystem level, which emphasizes the dynamic interplay between different microsystems. From this viewpoint, job stress arising in the exosystem may undermine marital quality, and the resulting strain may limit teachers’ capacity to mobilize social support from colleagues, friends, or family members. This sequential erosion of relational resources may ultimately reduce well-being, pointing to a possible chain mediation mechanism that warrants empirical examination.

## The current study

**Figure 1:**
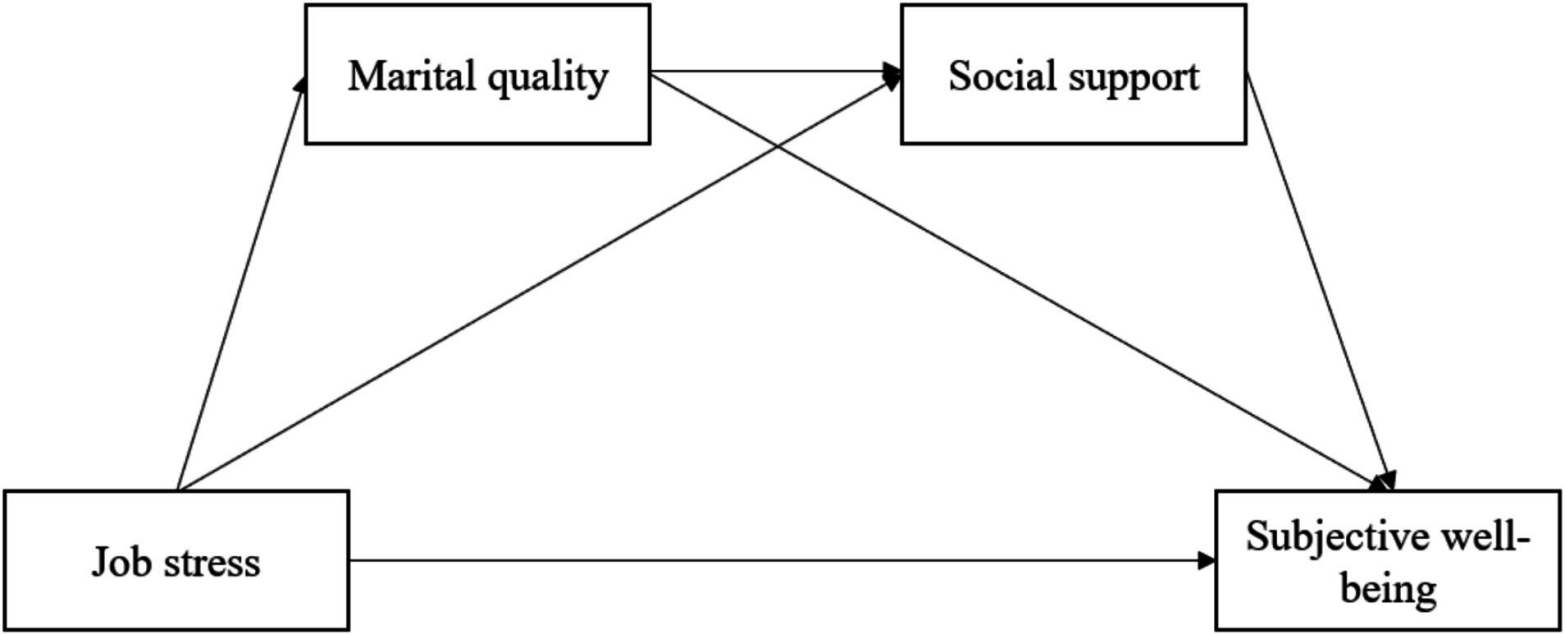
Proposed sequential mediation model.

The present study investigates the relationships among job stress, marital quality, social support, and subjective well-being among Chinese teachers. Based on the ecological systems theory (Bronfenbrenner, 1979, 2005), we constructed a sequential mediation model to examine the marital quality and social support’s indirect role in the relationship between job stress and subjective well-being, as illustrated in Figure 1. This study tests the following hypotheses:

*Hypothesis 1*: Teachers’ job stress is negatively associated with subjective well-being.

*Hypothesis 2*: Marital quality mediates the relationship between teachers’ job stress and subjective well-being.

*Hypothesis 3*: Social support mediates the relationship between teachers’ job stress and subjective well-being.

*Hypothesis 4*: Marital quality and social support sequentially mediate the relationship between teachers’ job stress and subjective well-being.

This study aims to identify key interpersonal resources related to teachers’ well-being and to clarify the mechanisms linking job stress to SWB. To extend the applicability of ecological systems theory by highlighting how exosystem stressors interact with microsystem resources, both independently and sequentially. The findings are expected to provide evidence-based recommendations for enhancing teacher support systems and promoting sustainable well-being in the Chinese educational context.

## Methods

### Participants

The present study employed a convenience sampling approach to recruit 251 elementary and secondary school teachers from Shandong province and Xinjiang Uygur Autonomous Region, China. Approximately 280 teachers were initially invited (via online invitations and school contacts), and 251 of them provided valid responses, yielding a response rate of about 90%. This sampling method was chosen for its practicality and feasibility in accessing the target population (i.e., school teachers in the targeted regions). As the study focused on married participants, 62 unmarried teachers were excluded, yielding a final analytic sample of 189 married teachers. Only married teachers were asked to complete the marital quality scale; accordingly, data from unmarried respondents were excluded from all analyses. Table 1 presents the demographic characteristics of the sample. A total of 189 teachers participated in the study, comprising 107 males (56.6%) and 82 females (43.4%). Most participants were between 31 and 50 years of age (71.4%), with 33.3% aged 31–40 years and 38.1% aged 41–50 years. In terms of educational attainment, the majority held a bachelor’s degree (83.6%), followed by vocational college (10.6%), a master’s degree (4.2%), a high school diploma or lower (1.1%), and doctoral degrees (0.5%). Regarding teaching level, 64.6% taught at the primary level, while 35.4% were secondary school teachers. (All participants were full-time teachers at the time of the survey.)

**Table 1:** Demographic characteristics of the participants (*N* = 189).

Variable	Category	Frequency (*n*)	Percentage (%)	*M (SD)*
Gender	Male	107	56.6	1.433 (0.497)
	Female	82	43.4	
Age	18–25	6	3.2	3.508 (0.976)
	26–30	20	10.6	
	31–40	63	33.3	
	41–50	72	38.1	
	51–60	28	14.8	
Education levels	High school or below	2	1.1	2.926 (0.455)
	Vocational college degree	20	10.6	
	Bachelor’s degree	158	83.6	
	Master’s degree	8	4.2	
	Doctoral degree	1	0.5	
Teaching level	Primary	122	64.6	1.355 (0.480)
	Secondary	67	35.4	

### Procedures

Before the commencement of the survey, the research protocol underwent a comprehensive ethical review by the first author’s academic institution. The review process considered aspects such as informed consent, confidentiality, and the potential risks and benefits of the study. With the assistance of local education bureaus, the Wenjuan Xing online questionnaire was distributed to teachers in primary and secondary schools across Shandong and Xinjiang provinces, who completed and submitted it anonymously. This platform offered several advantages, including ease of administration, efficient data collection, and the ability to reach a geographically dispersed sample. All participating teachers provided their voluntary informed consent to take part in the study. The questionnaire collected demographic information (e.g., teacher gender, age, marital status) and included a series of scales measuring teachers’ job stress, well-being, marital quality, and social support. The survey link remained open for approximately 1 week. All respondents completed the questionnaire with sections presented in a fixed order (demographic questions first, followed by the job stress, well-being, social support, and marital quality scales). Participants were assured that their responses would remain confidential and anonymous. Furthermore, all data may be shared in an anonymized format for research purposes only, ensuring both transparency and protection of participant privacy.

### Measures

#### Teacher job stress

The Teacher Work Stress Questionnaire used in this study was developed and validated by Shi et al. (2005). The instrument consists of 53 items, including 36 items that measure *sources of stress* and 17 items that assess *stress responses*. Participants rated their perceived work-related stress on a 5-point Likert scale ranging from 1 (“no stress”) to 5 (“very high stress”). The total score ranges from 53 to 265, with higher scores indicating greater work-related stress. This study employed the validated Chinese version of the scale, which has demonstrated good criterion validity (Shi et al., 2005). In the current sample, the overall internal consistency was excellent (Cronbach’s *α* = 0.986). The two subscales also showed very high reliability, with Cronbach’s *α* = 0.982 for stress sources and *α* = 0.965 for stress responses.

#### Teacher’s general well-being

The Chinese version of the General Well-Being Schedule (GWBS), originally developed by Fazio (1977) and translated by Duan (1996), was used to assess teachers’ subjective well-being. This Chinese adaptation has been validated in prior studies, showing high internal consistency (Cronbach’s *α* = 0.90; Huang et al., 2023). The scale comprises 18 items measuring six dimensions: satisfaction and interest in life, health concern, energy level, cheerful mood, emotional and behavioral control, and relaxation and tension. Fourteen items are rated on a 6-point scale and four items on a 10-point scale, yielding a total score ranging approximately from 18 to 124. The total GWBS score was used to represent overall well-being, with higher scores indicating greater subjective well-being. Several negatively worded items (e.g., those reflecting depressed mood) were reverse-scored to ensure that higher scores consistently signified higher well-being. In the present study, the GWBS demonstrated good internal consistency (Cronbach’s *α* = 0.822).

#### Teacher social support

The Chinese version of the Multidimensional Scale of Perceived Social Support (MSPSS) was initially developed by Chou (2000) in Hong Kong and later adapted into simplified Chinese by Guan et al. (2013). Both studies provided evidence supporting the reliability and validity of the Chinese adaptation. The scale includes 12 items divided into three dimensions: family, friends, and significant other. Each item is rated on a 7-point Likert scale from 1 (“very strongly disagree”) to 7 (“very strongly agree”). Higher scores indicate greater perceived social support. Subscale scores are calculated by summing the four items within each dimension (range = 4–28), and the total MSPSS score ranges from 12 to 84. In the present study, the MSPSS demonstrated excellent internal consistency (Cronbach’s *α* = 0.954). Each of the three subscales also showed high reliability: family support (*α* = 0.909), friend support (*α* = 0.911), and significant-other support (*α* = 0.900). These results are consistent with prior findings indicating that the Chinese MSPSS exhibits strong reliability (*α* = 0.85–0.91) and a robust three-factor structure across diverse populations.

#### Marital quality

The Marital Quality Questionnaire developed by Olson et al. (1983) was used to assess participants’ marital quality. We employed the Chinese adaptation of this instrument (the ENRICH Marital Inventory), which has demonstrated high internal reliability (Cronbach’s *α* ranging from approximately 0.69 to 0.97 across subscales) and strong construct validity in previous studies (Guan et al., 2013). The questionnaire comprises 124 items across 12 dimensions: idealization, marital satisfaction, personality compatibility, spousal communication, conflict resolution, financial management, leisure activities, sexual relationship, children and marriage, relationships with relatives and friends, role equality, and value/religious agreement. All dimensions except idealization include 10 items, whereas the idealization dimension contains 14 items. Each item is rated on a 5-point Likert scale ranging from 1 (“definitely true”) to 5 (“definitely not true”), with higher scores indicating better marital quality. Negatively phrased items were reverse-coded so that higher scores consistently reflect better perceived marital quality. Following the standard scoring convention for this instrument, the idealization dimension is often treated as a response-bias indicator. It was not included in the computation of the total marital quality score or in the calculation of Cronbach’s *α*. Accordingly, total scores were derived from the remaining 11 subscales. Since each of these 11 subscales contains 10 items scored from 1 to 5, the total marital quality score could range from 110 to 550, with higher scores representing better overall marital quality. In the present study, the questionnaire demonstrated good internal consistency for the total score (Cronbach’s *α* = 0.903).

### Analytic approaches

The data analysis proceeded in three stages. In the first stage, descriptive statistics and Pearson correlations were computed for the primary study variables, including teacher job stress, subjective well-being, social support, and marital quality. The distributional assumptions were also assessed by examining skewness and kurtosis values. Moreover, to examine the presence of common method bias, Harman’s single-factor test was conducted. In the second stage, multiple regression analysis was conducted in SPSS 26 to explore the direct effect of teacher job stress on subjective well-being. In the third stage, structural equation modeling (SEM) was employed to investigate whether marital quality and social support sequentially mediated the relationship between teacher job stress and subjective well-being (see Figure 1). Consistent with Hayes (2018), a bootstrap procedure was used to test the significance of indirect effects, as this method yields more accurate estimates than conventional significance tests. Specifically, 5,000 bootstrap samples were generated to calculate point estimates and 95% bias-corrected confidence intervals. Indirect effects were considered significant if the confidence interval did not include zero. As all models were saturated (i.e., just-identified), model fit indices were not reported (Kline, 2016). All descriptive and correlational analyses were performed in SPSS 26, whereas SEM was conducted using Mplus 8.3.

## Results

### Common method bias, descriptive statistics, and correlations

To reduce the likelihood of common method bias, this study utilized response tokens and ensured anonymity in participants’ answers (Zhou and Long, 2004). In addition, Harman’s single-factor test was employed as a diagnostic procedure (Podsakoff et al., 2003). The exploratory factor analysis revealed that the first factor explained 20.06% of the total variance, which is substantially lower than the recommended threshold of 40%. This result indicates that common method variance was unlikely to pose a significant threat in the present study. Furthermore, according to Hair et al. (2010), data can be considered approximately normal when skewness values range between −2 and +2 and kurtosis values between −7 and +7. Consistent with this guideline, all study variables exhibited an acceptable level of normality, with skewness and kurtosis both remaining within |2|.

Table 2 presents the descriptive statistics and zero-order correlations among all study variables. Additionally, to examine the relationships among the key variables, we generated scatter plots for all pairwise combinations of the four variables in this study (Supplementary Figures S1–S6). Job stress was negatively correlated with marital quality (*r* = −0.333, *p* < 0.001) and subjective well-being (*r* = −0.685, *p* < 0.001), indicating that higher levels of job stress were associated with poorer marital relationships and lower subjective well-being. Marital quality was positively related to social support (*r* = 0.228, *p* < 0.01) and subjective well-being (*r* = 0.531, *p* < 0.001), suggesting that teachers with higher marital quality tended to report greater social support and well-being. Social support also showed a significant positive association with subjective well-being (*r* = 0.201, *p* < 0.01).

**Table 2:** Descriptive statistics and zero-order correlations for all variables (*N* = 189).

Variable	Scoring range	1	2	3	4
1. Job stress	53–265	**–**			
2. Marital quality	243–453	−0.333^***^	**–**		
3. Social support	12–84	−0.009	0.228^**^	**–**	
4. Subjective well-being	34–116	−0.685^***^	0.531^***^	0.201^**^	**–**
*M* (*SD*)		160.471 (47.394)	339.519 (35.219)	55.804 (14.573)	75.646 (14.243)
*Skewness*		−0.440	0.992	−0.295	0.263
*Kurtosis*		0.186	1.549	0.661	0.724

^*^p < 0.05, ^**^p < 0.01,^***^p < 0.001.

### The direct role of the teachers’ job stress on subjective well-being

As presented in Table 3, the results of hierarchical regression analysis found that teachers’ job stress significantly and negatively predicted subjective well-being (*β* = −0.685, *p* < 0.001) in Step 1. This result was consistent with our hypothesis and indicated that higher levels of job stress were associated with lower levels of subjective well-being. In Step 2, marital quality (*β* = 0.309, *p* < 0.001) and social support (*β* = 0.125, *p* < 0.05) were added to the model, both of which significantly and positively predicted teachers’ subjective well-being. These findings suggest that teachers with higher marital quality and stronger social support tend to report greater well-being, even after controlling for job stress.

**Table 3:** Regression analysis to predict subjective well-being.

Step	Predictor variable	*R*^2^	*F*	*β*	*SE*	*t*	*VIF*
Step 1	Job stress	0.469	164.951***	−0.685***	0.016	−12.843	1.000
Step 2	Job stress	0.587	87.524***	−0.581***	0.015	−11.549	1.131
	Marital quality			0.309***	0.021	5.987	1.193
	Social support			0.125*	0.048	2.572	1.060

*β* refers to standardized regression coefficients. *p < 0.05, **p < 0.01, ***p < 0.001.

Moreover, adding marital quality and social support in Step 2 explained an additional 11.8% of the variance in teachers’ subjective well-being, resulting in a total of 58.7% of the variance being explained by the full model. In addition, all variance inflation factor (VIF) values ranged from 1.000 to 1.193, well below the recommended threshold of 5 (Kim, 2019), indicating no multicollinearity issues among the predictors.

### The indirect role of marital quality and social support

**Figure 2:**
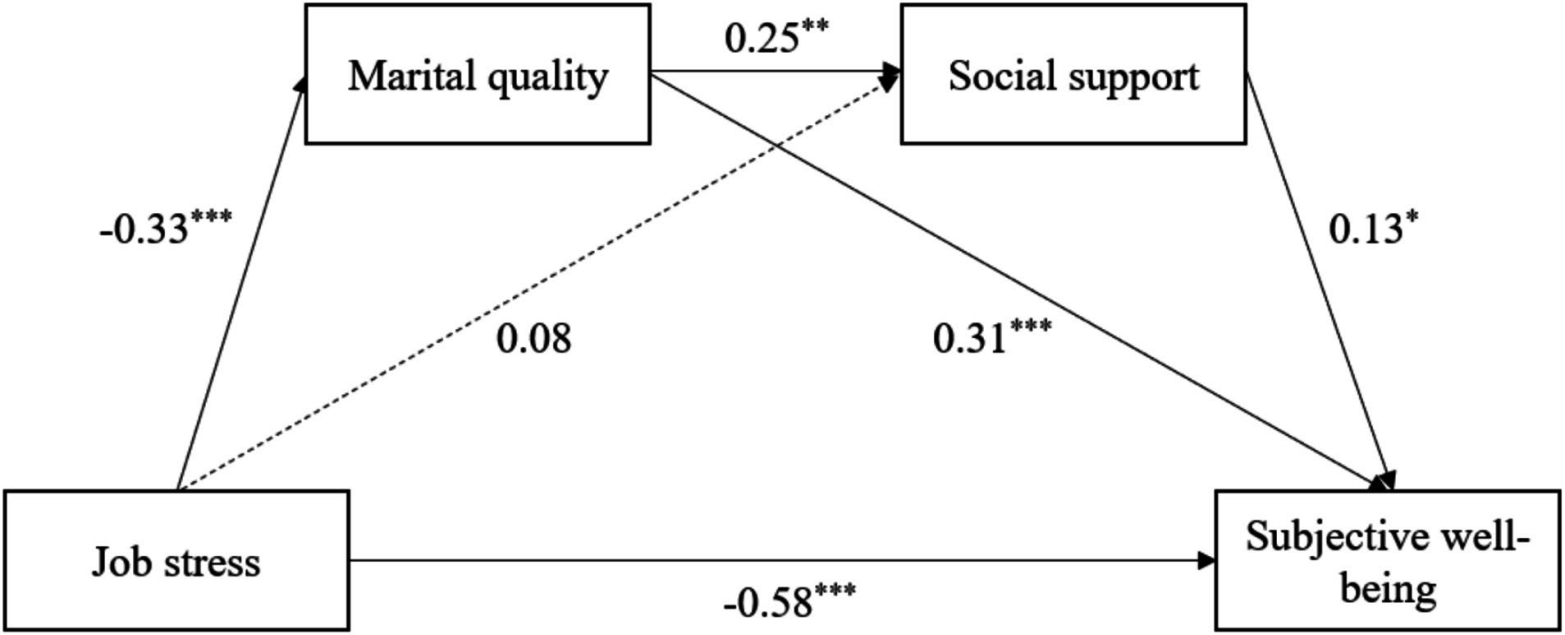
The mediation role of marital quality and social support between job stress and subjective well-being. The path values represent standardized estimates. *p < 0.05, **p < 0.01, ***p < 0.001.

As illustrated in Figure 2, we further explored a sequential mediation model in which marital quality predicted social support. The results showed that job stress was significantly associated with marital quality (*β* = −0.33, *p* < 0.001), which in turn positively predicted social support (*β* = 0.25, *p* < 0.01), and social support significantly predicted teachers’ subjective well-being (*β* = 0.13, *p* < 0.05). Also, the marital quality was significantly and positively linked to teachers’ subjective well-being (*β* = 0.31, *p* < 0.001). Moreover, job stress was significantly and negatively associated with teachers’ subjective well-being (*β* = −0.58, *p* < 0.001). Notably, teachers’ job stress was not associated with social support, which indicated that social support did not mediate the relationship between teachers’ job stress and subjective well-being.

As presented in Model 1 of Table 4 and in line with Hypothesis 2, the bootstrap-corrected confidence intervals for the indirect role of marital quality in the relationship between teachers’ job stress and subjective well-being were significant (indirect effect = −0.103, *SE* = 0.030, 95% *CI* [−0.169, −0.053]). The confidence interval excluded zero, indicating that marital quality plays a partial mediating role in the association between the teachers’ job stress and subjective well-being. Similarly, as shown in Model 3 of Table 4 and in line with Hypothesis 4, marital quality and social support in the relationship between teachers’ job stress and subjective well-being were significant (indirect effect = −0.011, *SE* = 0.007, 95% *CI* [−0.026, −0.001]). The confidence interval excluded zero, indicating that marital quality and social support play a partial sequential mediating role in the association between teachers’ job stress and subjective well-being.

**Table 4:** Mediation analysis of the models linking job stress and subjective well-being.

Path	Effect	SE	LL 95% CI	UL 95% CI
Model 1 Job stress→Marital quality→Subjective well-being	−0.103 *^a^*	0.030	−0.169	−0.053
Model 2 Job stress→Social support→Subjective well-being	0.009	0.015	−0.019	0.044
Model 3 Job stress→Marital quality→Social support→Subjective well-being	−0.011 *^a^*	0.007	−0.026	−0.001

LL 95% CI = lower limit of the 95% confidence interval; UL 95% CI, upper limit of the 95% confidence interval. ^**a**^Bootstrap 95% CI does not include 0.

## Discussion

This study explored how teachers’ job stress related to their subjective well-being within the Chinese educational context. The results suggest that subjective well-being is related to teachers’ job stress and the quality of their interpersonal relationships (e.g., marital quality and social support). By identifying both direct and indirect pathways, the study underscores the complex and multi-layered processes through which stress is linked to subjective well-being, providing theoretical support for ecological systems theory and highlighting the interconnected roles of school, family, and social systems.

### The direct effect of teachers’ job stress on subjective well-being

This study found a significant negative association between teachers’ job stress and their subjective well-being. In other words, teachers experiencing higher levels of job stress reported lower levels of subjective well-being, thus supporting Hypothesis 1. This result is consistent with the findings of Shao et al. (2025) and Mérida-López et al. (2022), indicating that within the Chinese cultural and educational context, high job stress similarly reduces teachers’ life satisfaction and overall subjective well-being.

These findings provide empirical support for the ecological systems theory (Bronfenbrenner, 1979, 2005), which posits that proximal processes within the microsystem exert a direct influence on individual development. The school microsystem constitutes a critical setting where teachers’ stress is generated and accumulated. When teaching demands, administrative requirements, and emotional burdens persist within this system, they transform into chronic stressors that undermine teachers’ subjective well-being. This mechanism suggests that teachers’ subjective well-being is not determined solely by personal traits but is also profoundly shaped by their daily work environment. Therefore, this study demonstrates the direct role of the school microsystem related to teachers’ subjective well-being and highlights the importance of contextual factors in understanding variations in subjective well-being.

### The central role of marital quality and the limitations of social support

This study found that marital quality served as a significant mediator in the relationship between teachers’ job stress and subjective well-being. Specifically, higher job stress was associated with lower marital quality, which in turn led to reduced subjective well-being, thereby supporting Hypothesis 2. This finding provides empirical evidence for understanding how occupational stress is linked to subjective well-being. In contrast, social support did not mediate the relationship between job stress and subjective well-being, and Hypothesis 3 was not supported. This suggests that social support may not be the primary mechanism, whereas marital quality plays a more central role in linking teachers’ stress and well-being.

These findings provide empirical confirmation of ecological systems theory (Bronfenbrenner, 1979, 2005), particularly its emphasis on the interconnections between systems. Teachers’ job stress is not confined to the school microsystem; it also spills over into the family microsystem through cross-system “spillover effects” (Grzywacz and Marks, 2000), thereby undermining marital quality and reducing well-being. This process reflects the role of the mesosystem, highlighting how interactions between school and family systems are jointly related to teachers’ well-being. The findings underscore the importance of family systems, compensate for the lack of attention to family dynamics in prior research, and extend the applicability of ecological systems theory within the Chinese educational context.

### The sequential mediation of marital quality and social support

This study further revealed that teachers’ job stress is indirectly linked to well-being through the sequential mediating roles of marital quality and social support, thus supporting Hypothesis 4. Specifically, higher job stress reduced marital quality, which subsequently led to diminished social support, ultimately resulting in lower levels of well-being.

This finding validates and extends ecological systems theory (Bronfenbrenner, 1979, 2005) by illustrating the interdependence of multiple systems. Teachers’ stress within the school microsystem first undermines marital relationships in the family microsystem, which in turn weakens broader social support networks, thereby reducing subjective well-being. This sequential mediation pathway reveals a cross-system cascading effect, showing that a single system does not determine teachers’ well-being but is related to the dynamic interconnections among school, family, and social systems. This mechanism resonates with ecological systems theory’s emphasis on nested systems and interdependence (Neal and Neal, 2013) and enriches our understanding of how stress transmits across systems.

Furthermore, the findings indicate that stress experienced in one system not only depletes resources within that system but also triggers cross-system transmission effects that erode other relational resources, thereby producing cumulative negative impacts on well-being. Thus, the identified sequential mediation pathway extends the theoretical application of ecological systems theory in educational contexts and deepens our understanding of the mechanisms shaping teachers’ subjective well-being.

### Limitations and implications

Although this study revealed important relationships among teachers’ job stress, marital quality, social support, and subjective well-being, several limitations should be noted. First, the use of a cross-sectional design limits the ability to draw causal inferences among variables. Future studies should employ longitudinal or experimental designs to further examine the dynamic processes linking stress, relational resources, and well-being. Second, the sample was drawn from teachers in Shandong and Xinjiang Provinces, which may limit the representativeness of the findings for teachers nationwide. Moreover, a non-probabilistic (convenience) sampling method was used, which may further restrict generalizability. Future studies should consider using larger and more diverse samples across multiple regions to enhance the generalizability of the results. Third, the study relied on self-report questionnaires. Although efforts were made to ensure the reliability and validity of the measures, the data may still be subject to social desirability effects. Fourth, the study primarily focused on the mediating roles of marital quality and social support. At the same time, other potential factors were not considered, such as teachers’ personality traits, organizational climate, or cultural variables. Future studies could also include relevant covariates (e.g., teachers’ years of teaching experience and school type) to better account for contextual influences on teachers’ subjective well-being. Fifth, although SEM was used to examine the hypothesized mediation model, all constructs were treated as observed variables rather than latent factors. Given the modest sample size (*N* = 189) and the large number of model parameters (over 20 dimensions across all variables), this approach ensured model stability but limited the control of measurement error. Prior research suggests that the ratio of sample size to estimated parameters should not fall below 10:1 for stable latent-variable estimation (Jackson, 2003). Future research with larger samples should employ full latent-variable SEM to yield more accurate and robust results.

Despite these limitations, the findings yield several important implications for educational practice in China. First, enhancing teachers’ well-being requires coordinated efforts across multiple levels. Schools and educational authorities should mitigate the negative effects of job stress on teachers’ well-being by redistributing teaching and administrative tasks more equitably, streamlining formalities, and offering professional development opportunities along with psychological support (Hartney, 2016; Keeley, 2023). Given the cultural context of China, where family and marital quality play a pivotal role in teachers’ well-being, policies should also promote work–family balance. Initiatives such as flexible scheduling, paid leave, and access to family counseling services (Cvenkel, 2020; Akhter and Mahmud, 2025) could help mitigate the “spillover effects” of occupational stress. At the same time, schools should go beyond superficial collegial support to build deeper support systems, such as mentoring programs, peer support groups, and stronger home–school collaboration, to enhance teachers’ sense of social support (Chelelwa et al., 2025; Putra et al., 2024).

More importantly, improving teachers’ well-being requires multi-level and integrated interventions. At the individual level, mindfulness training and stress management programs can strengthen teachers’ self-regulation abilities (Rana et al., 2022; Sehgal and Kaur, 2024). At the family level, fostering communication and relational support is crucial for maintaining family functioning. At the societal level, establishing community-based and union-based support networks can help prevent the erosion of relational resources. The coordinated implementation of these multidimensional measures may alleviate teachers’ job stress, enhance their subjective well-being, and ultimately contribute to greater professional engagement and improved educational quality.

## Conclusion

This study demonstrates that teachers’ job stress significantly undermines their subjective well-being both directly and indirectly through relational pathways. Marital quality emerged as a central mediator, while marital quality and social support functioned sequentially, revealing cross-system processes through which stress cascades from school to family and broader social networks. These findings extend ecological systems theory by showing how the interdependence of school, family, and social contexts shapes teachers’ well-being. Beyond theoretical contributions, the results suggest that multi-level strategies addressing school, family, and social systems are essential for improving teachers’ well-being.

## Data Availability

The raw data supporting the conclusions of this article will be made available by the authors, without undue reservation.
