# Supplementary material for: The job stress and subjective well-being among Chinese primary and secondary school teachers: the role of marital quality and social support
Source: Front Psychol. 2026 Jan 21;16:1625960. doi: 10.3389/fpsyg.2025.1625960 (PMC12869313; doi:10.3389/fpsyg.2025.1625960)
Supplement: Supplementary file 1 [file Data_Sheet_1.docx]

**The job stress and subjective well-being** **among Chinese primary and secondary school teachers: the role of marital quality and social support**

**Supplementary Materials**


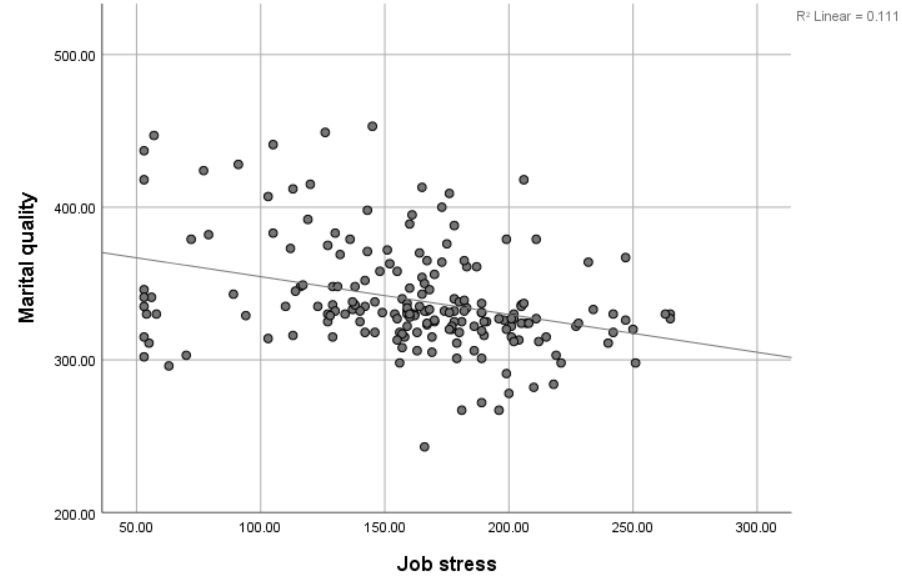


Figure S1. Scatter Plot of the Relationship Between Job Stress and Marital Quality with a Linear Trend Line


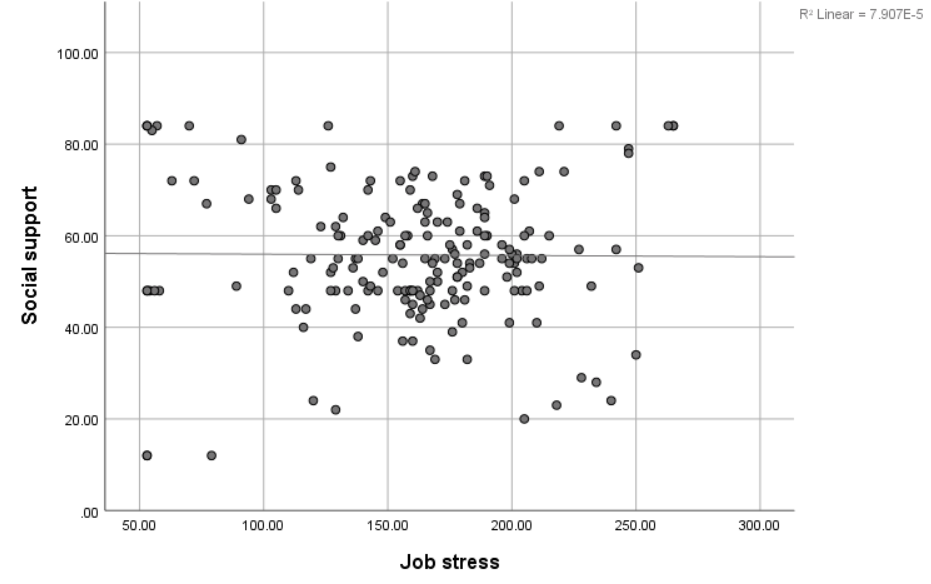


Figure S2. Scatter Plot of the Relationship Between Job Stress and Social Support with a Linear Trend Line


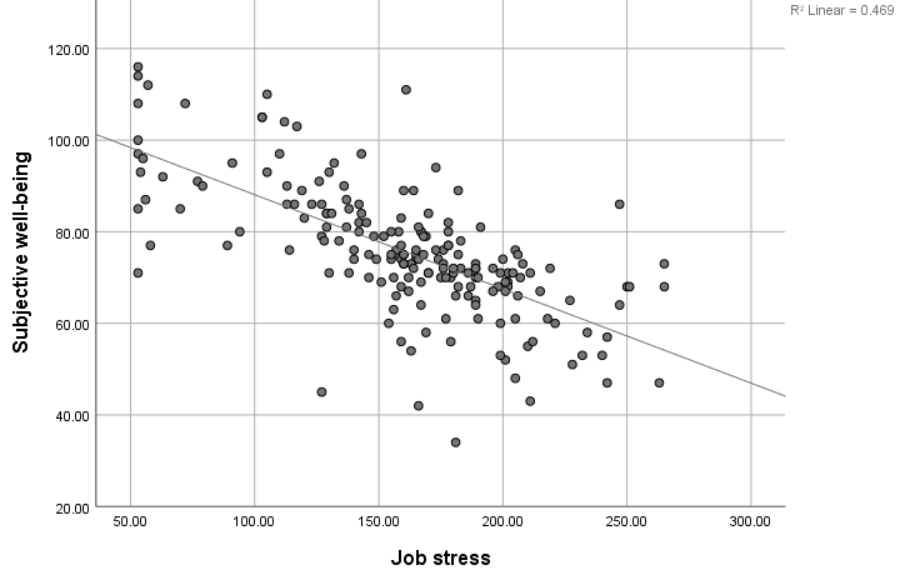


Figure S3. Scatter Plot of the Relationship Between Job Stress and Subjective Well-being with a Linear Trend Line


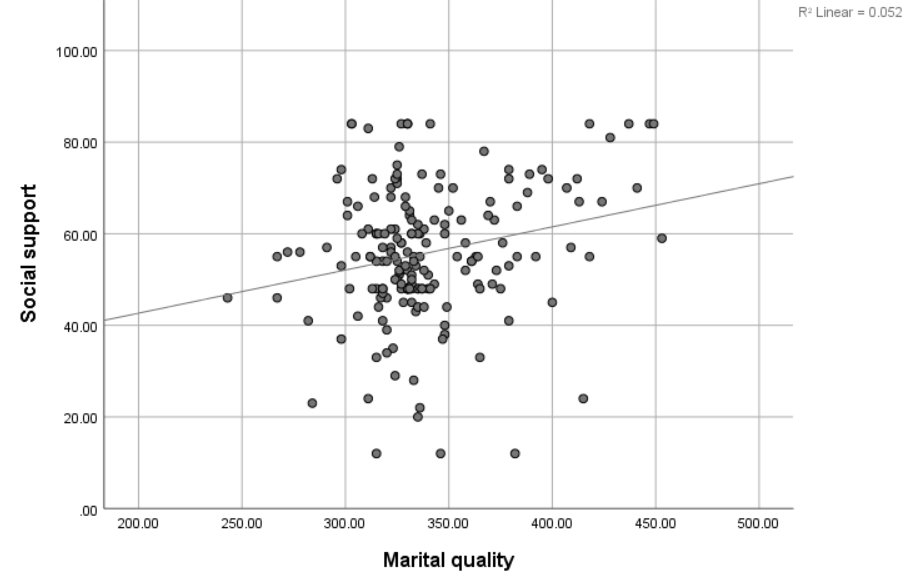


Figure S4. Scatter Plot of the Relationship Between Marital Quality and Social Support with a Linear Trend Line


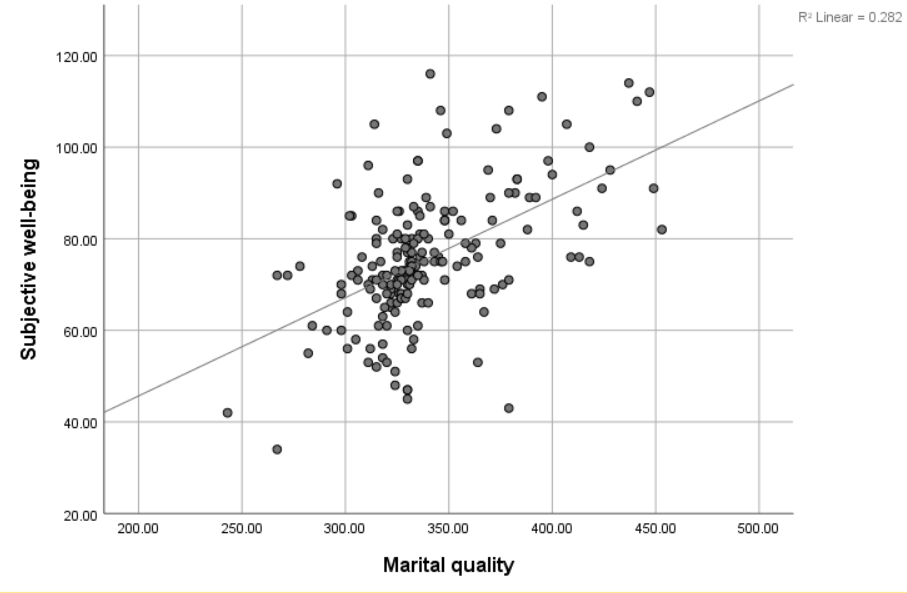


Figure S5. Scatter Plot of the Relationship Between Marital Quality and Subjective Well-being with a Linear Trend Line


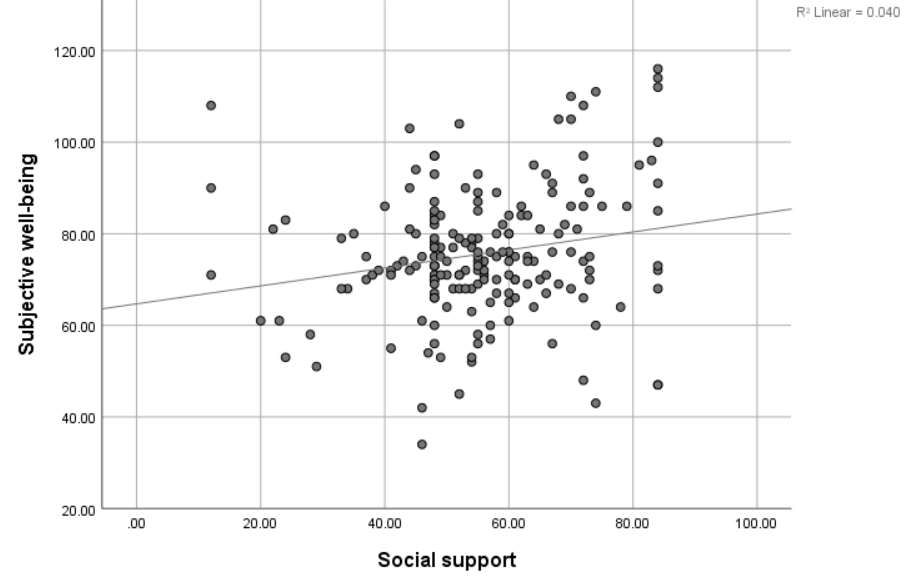


Figure S6. Scatter Plot of the Relationship Between Social Support and Subjective Well-being with a Linear Trend Line
